# Analysis and Applications of Neuromorphic Memristors in Artificial Intelligence Computing

**DOI:** 10.1007/s40820-026-02322-5

**Published:** 2026-08-19

**Authors:** Jianhui Wang, Qingxin Chen, Jinhao Zhang, Jialin Meng, Tianyu Wang

**Affiliations:** 1 School of Integrated Circuits, Shandong Key Laboratory of Next-Generation Semiconductor Technology and Systems, Shandong University, Jinan, 250100 People’s Republic of China; 2 Suzhou Research Institute of Shandong University, Suzhou, 215123 People’s Republic of China; 3 National International Innovation Center, Shanghai, 201203 People’s Republic of China; 4 Key Laboratory of Computational Neuroscience and Brain-Inspired Intelligence (Fudan University), Ministry of Education, Shanghai, 200433 People’s Republic of China; 5 State Key Laboratory of Crystal Materials, Shandong University, Jinan, 250100 People’s Republic of China

**Keywords:** Neuromorphic memristors, Neuromorphic computing, Artificial intelligence, In-memory computing

## Abstract

Artificial intelligence (AI) has advanced rapidly in recent years and has been widely applied in healthcare, intelligent sensing, machine perception, and image recognition. Neuromorphic computing, inspired by the structure and operating principles of the human brain, has emerged as a promising paradigm for building efficient, low-power, and adaptive information processing systems. In this review, we summarize the development of neuromorphic memristors from the perspectives of biological inspiration, representative material systems, device architectures, performance metrics, and artificial intelligence-oriented applications. Different from previous reviews that mainly focus on memristor materials, switching mechanisms, or neuromorphic functions separately, this article further emphasizes the relationships between memristor characteristics and distinct AI-oriented tasks, including AI acceleration, neuromorphic computing, intelligent sensing, and human–machine interaction. Finally, the major challenges and future opportunities of neuromorphic memristors are discussed from the viewpoints of device optimization, system integration, scalability, and practical application.

## Hightlights

- Task-oriented perspective on neuromorphic memristors for artificial intelligence computing.
- From material systems and device structures to applications, flexibility, and system integration.
- A critical roadmap toward scalable, manufacturable, and practical neuromorphic hardware.

## Graphical abstract

**Figure:**
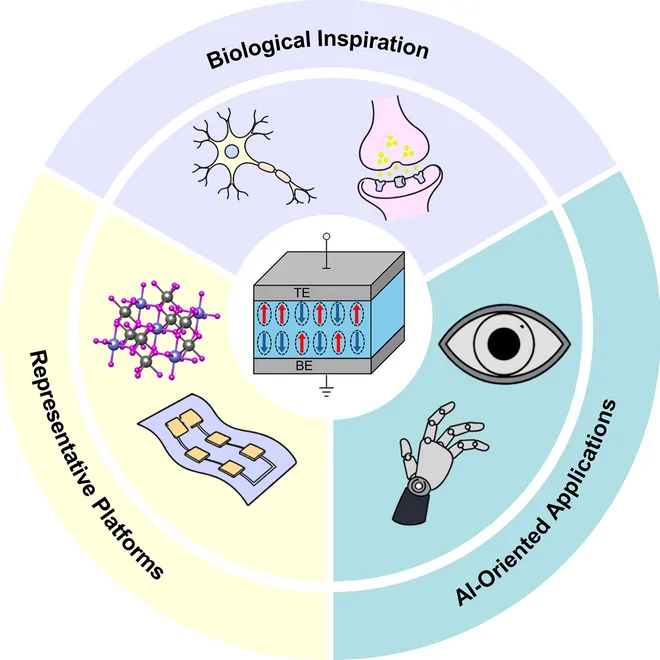

## Introduction

Driven by the continuous transistor miniaturization predicted by Moore’s law, the performance of CMOS-based computers has improved significantly over the past several decades [1, 2]. However, the rapid growth of data and increasingly complex computational tasks have exposed the limitations of the traditional von Neumann architecture. At the device level, transistor scaling is approaching physical limits and causes performance degradation and reliability issues. At the architectural level, the physical separation of memory and processing leads to high data transfer energy consumption and severe latency, which is commonly referred to as the “memory wall” [3, 4]. Therefore, new devices and computing paradigms are urgently needed to improve energy efficiency and reduce data movement overhead in modern computing systems [5].

Neuromorphic computing is considered one of the most promising approaches for addressing the von Neumann bottleneck and developing next-generation computing systems. Foundational concepts of neuromorphic electronic systems were established by Mead, who proposed silicon hardware that emulates biological neural architectures and information processing principles [6]. In biological systems, information transmission relies on neurons and synapses, which operate in an event-driven, parallel, and highly energy-efficient way [7–9]. Inspired by these characteristics, memristors have attracted great attention as candidate hardware platforms for neuromorphic computing because their conductance states can be continuously modulated and retained, enabling the emulation of neuronal and synaptic behaviors [1, 10]. Owing to their structural simplicity, non-volatility, and compatibility with dense integration, neuromorphic memristors are considered highly promising for building low-power and adaptive computing systems [11, 12].

In recent years, neuromorphic memristors have also shown strong potential in broader intelligent hardware scenarios, including AI acceleration, intelligent sensing, machine perception, wearable electronics, and human–machine interaction. In these applications, different material systems and device architectures may offer distinct advantages in flexibility, power consumption, reliability, integration density, and multifunctionality [13–15]. Therefore, a systematic review that not only summarizes representative memristor platforms but also clarifies their relevance to different artificial intelligence-oriented tasks is highly desirable.

**Fig. 1:**
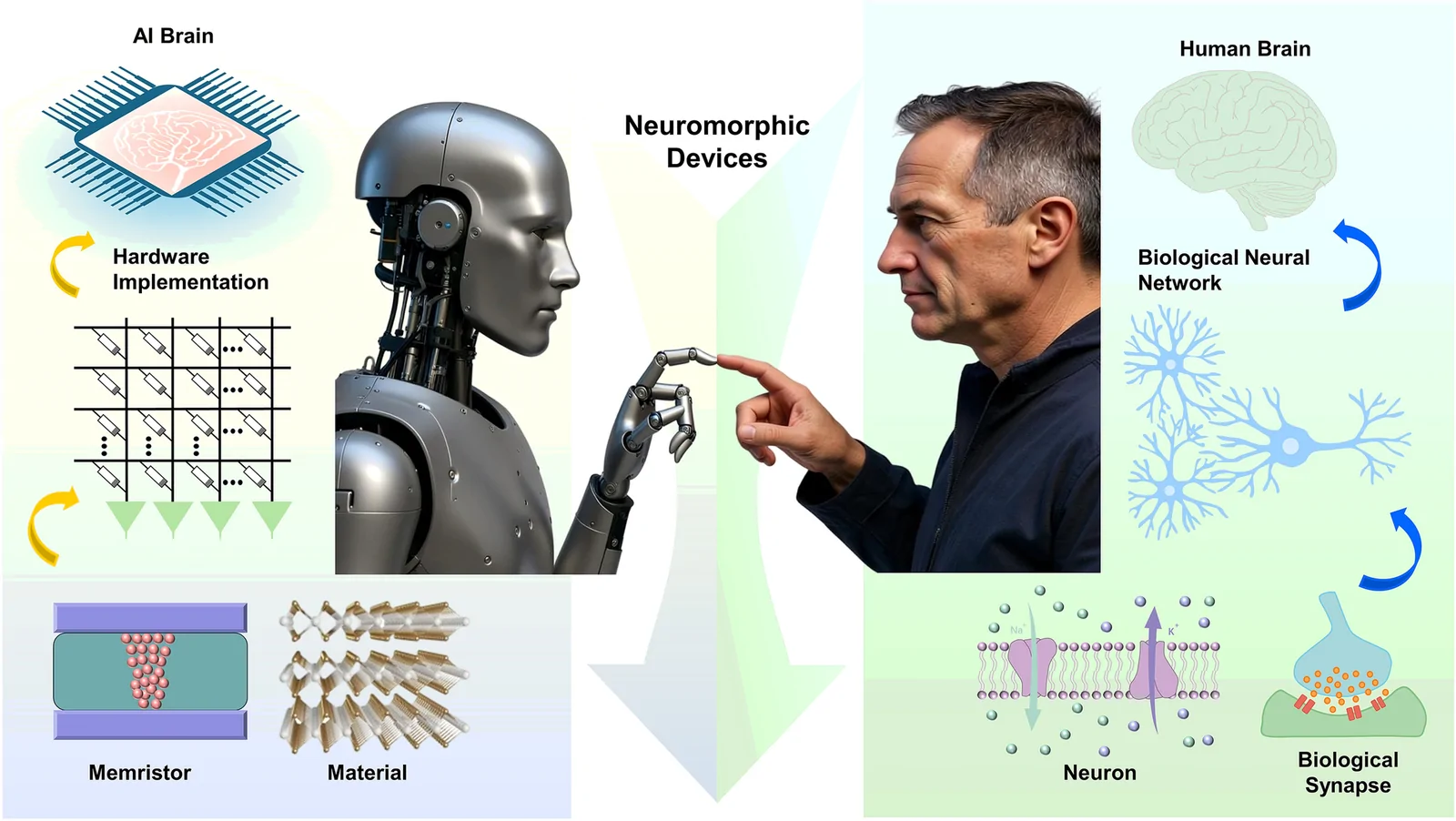
Ion movement is the foundation of information processing in biological synapses, neurons, and neural networks. Inspired by the biological nervous system, neuromorphic memristors aim to have an architecture and principles similar to biological nervous systems, promoting the development of artificial intelligence technology

This review focuses on neuromorphic memristors for artificial intelligence computing, with particular emphasis on the relationships among material systems, device architectures, performance metrics, and application scenarios [16]. Compared with previous reviews that mainly summarize memristor materials, switching mechanisms, or neuromorphic functions separately, this article further highlights how different memristor platforms support distinct AI-oriented tasks, including AI acceleration, neuromorphic computing, intelligent sensing, and human–machine interaction [17–19]. The manuscript is organized as follows. First, the biological inspiration and neural network relevance of neuromorphic computing are briefly introduced. Next, representative memristor platforms, device structures, and development history are summarized. Then, key performance metrics, including energy efficiency, reliability, flexibility, and system-level considerations, are discussed [20, 21]. Finally, representative application domains, critical bottlenecks, and future research directions are briefly outlined, as summarized in Fig. 1.

To clarify the positioning and novelty of the present review, Table 1 compares representative prior reviews related to memristors, neuromorphic computing, and AI hardware.

**Table 1:** Positioning of the present review relative to representative prior reviews

Year	References	Main focus	Limitation relative to the present review
2013	[138]	Memristive devices for computing	Early landmark review; published before the rapid development of AI-oriented memristor applications
2016	[173]	Synaptic electronics and neuromorphic computing	Focuses mainly on synaptic electronics, with less emphasis on broader AI-oriented hardware scenarios
2016	[174]	Memristors for energy-efficient computing paradigms	More paradigm-oriented, with less explicit task-based application mapping
2017	[175]	Neuromorphic computing using nonvolatile memory	Strong neuromorphic focus, but limited coverage of sensing, perception, and interaction applications
2017	[176]	Nanoionics-enabled memristive devices for neuromorphic applications	More material-/mechanism-centered than application-oriented
2018	[177]	Reconfigurable nanomaterials at the electronics–ionics interface	Important conceptual perspective, but not a broad review of AI-oriented neuromorphic memristors
2018	[178]	Future electronics based on memristive systems	More conceptual and forward-looking, with less systematic application classification
2019	[179]	Memristive crossbar arrays for brain-inspired computing	Focuses mainly on crossbar arrays and brain-inspired computing
2020	[180]	Resistive switching materials for information processing	Strong emphasis on materials and mechanisms rather than AI task-oriented system discussion
2020	[181]	Memory devices and applications for in-memory computing	Broader in-memory computing review, not specifically focused on neuromorphic memristors
2024	[182]	Memristor-based hardware accelerators for artificial intelligence	Strong accelerator-oriented review, with less emphasis on biological inspiration and broader neuromorphic applications
——	**This work**	**Neuromorphic memristors for artificial intelligence computing**	**Emphasizes the relationships among biological inspiration, representative memristor platforms, performance optimization, and distinct AI-oriented applications including AI acceleration, intelligent sensing, perception, and human–machine interaction**

As shown in Table 1, previous reviews have often focused on memristor materials and mechanisms, neuromorphic architectures, or AI accelerators separately. In contrast, the present review emphasizes the relationships among biological inspiration, representative memristor platforms, performance optimization, and distinct AI-oriented application scenarios, including AI acceleration, intelligent sensing, perception, and human–machine interaction.

## Biological Nervous System

This section briefly summarizes only those biological principles that are directly relevant to memristive neuromorphic hardware, including neuronal signal transmission, synaptic plasticity, and neural network-inspired information processing. Rather than providing a general overview of neurobiology, the discussion below focuses on the biological features that have most strongly inspired artificial synapses, artificial neurons, and neuromorphic computing systems. These include the conversion of external stimuli into electrical impulses, the transmission and temporal integration of neural signals, and the plastic adaptation of synaptic connections during learning.

### Human Nervous System

**Fig. 2:**
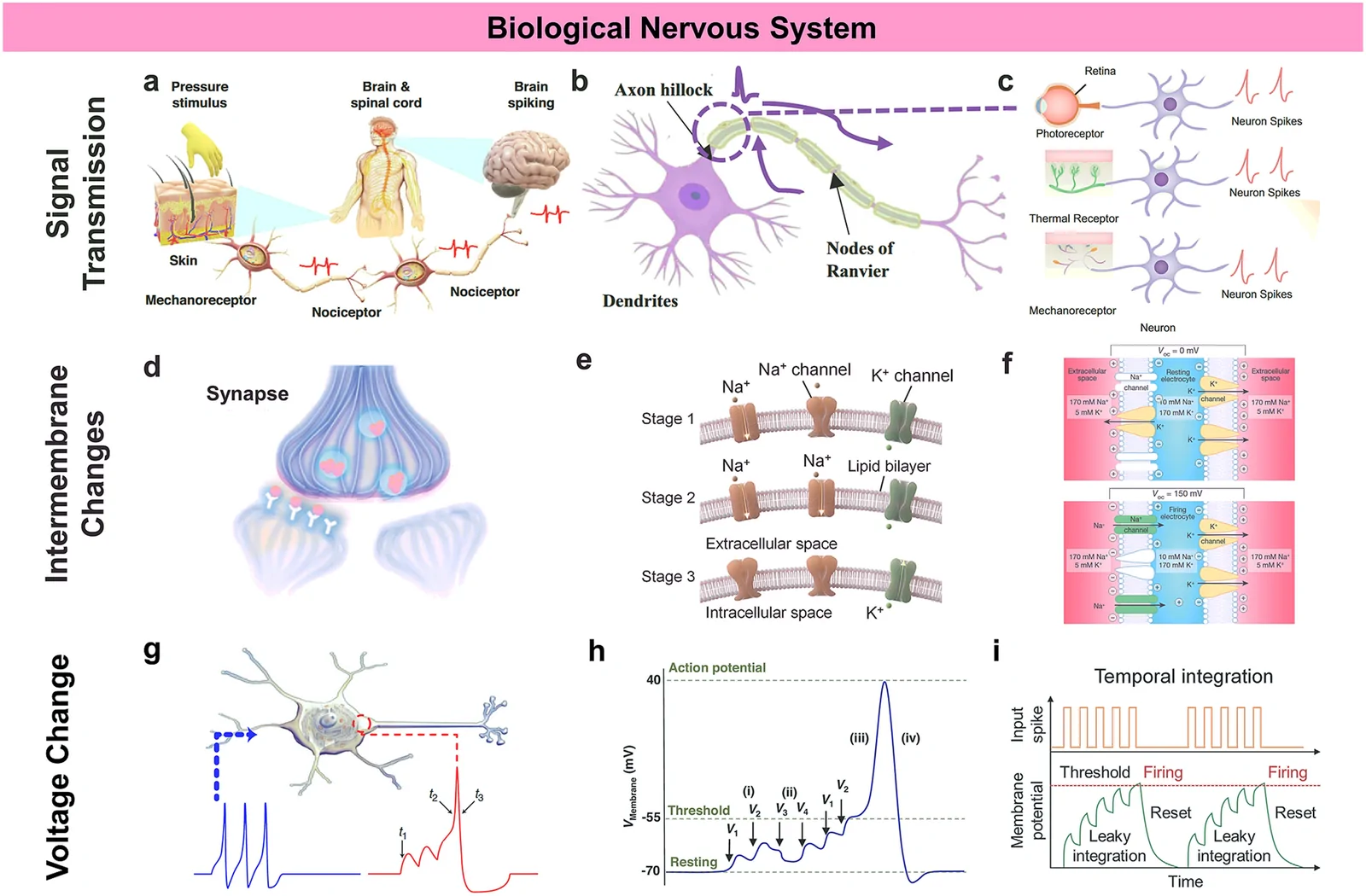
Biological nervous system. **a** Schematic diagram of the human nervous sensory system [83]. Reproduced with permission. Copyright 2025, Springer Nature. **b** Schematic structure of a neuron consisting of dendrites and axons [183]. Reproduced with permission. Copyright 2024, Springer Nature. **c** Receptors convert different external stimuli into electrical impulse signals [11]. Reproduced with permission. Copyright 2022, Springer Nature. **d** Specific structure of the synapse [184]. Reproduced with permission. Copyright 2024, John Wiley and Sons. **e** Ion activity between membranes causes changes in membrane potential [185]. Reproduced with permission. Copyright 2024, John Wiley and Sons. **f** Animals generate a cell potential of 150 mV through ion changes across membranes [3]. Reproduced with permission. Copyright 2017, Springer Nature. **g** Transmission of electrical impulse signals on neurons [10]. Reproduced with permission. Copyright 2018, Springer Nature. **h** Action potential generated after neuron stimulation [186]. Reproduced with permission. Copyright 2023, Springer Nature. **i** Membrane potential can be viewed as the temporal integration of input stimuli [185]. Reproduced with permission. Copyright 2024, John Wiley and Sons

In biological systems, information from the external environment is first detected by specialized receptors and then transmitted through neurons for further processing in the nervous system. From the perspective of neuromorphic hardware, the most relevant aspects are that external physical stimuli can be converted into electrical pulse signals and that neurons propagate these signals in an event-driven manner. As shown in Fig. 2a–c, photoreceptors, thermoreceptors, and mechanoreceptors transform different forms of environmental input into electrical impulses, thereby providing the biological basis for sensing and signal transmission in intelligent systems [22, 23].

Neurons are the fundamental processing units in this process. Their basic structure, including dendrites, the soma, and the axon, enables the reception, integration, and propagation of signals. When stimulation reaches a sufficient threshold, an action potential is generated near the axon hillock and propagates along the axon to downstream neurons. This event-driven signal transmission is highly energy-efficient and parallel in nature, which is one of the key reasons why neuronal systems have become a central source of inspiration for neuromorphic computing hardware [24, 25].

### Synapse

A synapse is the key functional junction for communication between neurons and is therefore one of the most important biological inspirations for memristive devices. Structurally, a synapse consists of the presynaptic terminal, the synaptic cleft, and the postsynaptic membrane. During signal transmission, neurotransmitters are released from the presynaptic terminal, diffuse across the synaptic cleft, and bind to receptors on the postsynaptic membrane, thereby inducing a postsynaptic response, as illustrated in Fig. 2d. This process is closely associated with ion channel activity and membrane potential modulation [26–28].

The generation of postsynaptic electrical responses depends on ion transport across the membrane. When stimulation arrives, Na⁺ channels open and allow Na⁺ to enter the cell, leading to membrane depolarization [29–31]. As the membrane potential rises above a threshold, Na⁺ channels become inactivated and K⁺ channels open, causing K⁺ efflux and subsequent repolarization, as shown in Fig. 2e–i. In this way, biological neurons integrate incoming signals over time and generate action potentials only when the accumulated input is sufficiently strong [32, 33]. Such temporal integration and threshold-triggered firing provide the direct biological basis for artificial neuron models and spike-based computing schemes [34, 35].

From the perspective of neuromorphic hardware, the most important biological features of synapses are analog weight modulation, temporal integration, and activity-dependent plasticity [36, 37]. In particular, synaptic learning rules such as spike-timing-dependent plasticity (STDP) and spike-rate-dependent plasticity (SRDP) are widely used as benchmarks for evaluating artificial synaptic devices. These biological processes are therefore introduced here not as general physiology, but as the functional foundation for understanding how memristive devices emulate excitatory postsynaptic current, paired-pulse facilitation, long-term potentiation/depression, and other learning-related behaviors [38, 39].

### Neural Networks

**Fig. 3:**
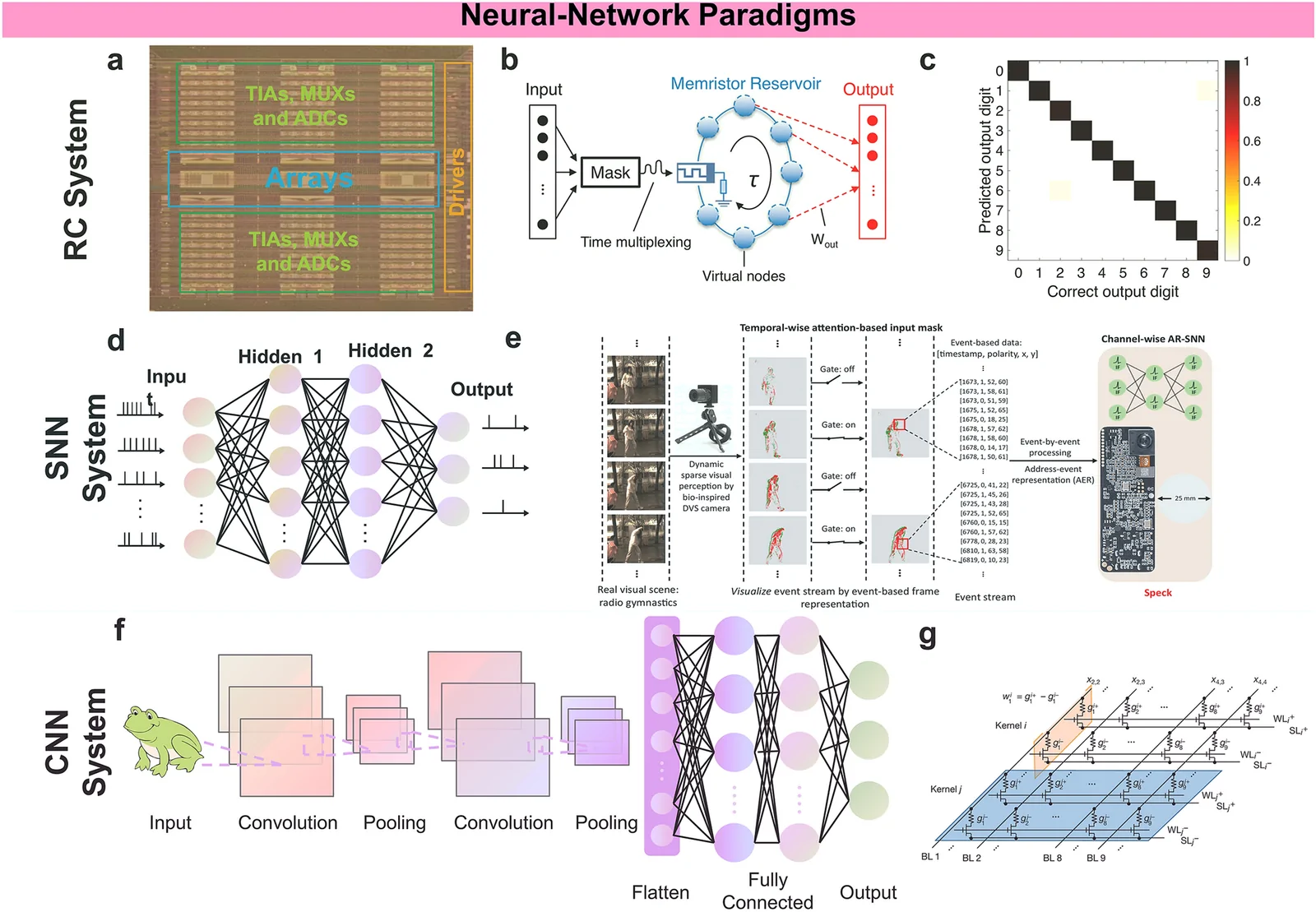
Representative neural network paradigms and their hardware relevance to memristive computing. **a** Optical image of a memristor-based neural network chip integrated with peripheral circuits [160]. Reproduced with permission. Copyright 2023, Springer Nature. **b** Schematic illustration of memristor-based reservoir computing [187]. Reproduced with permission. Copyright 2021, Springer Nature. **c** Recognition result of the reservoir system [187]. Reproduced with permission. Copyright 2021, Springer Nature. **d** Basic architecture of a spiking neural network [188]. **e** Event-driven visual processing and hardware implementation of an SNN system [188]. Reproduced with permission. Copyright 2024, Springer Nature. **f** Basic architecture of a convolutional neural network [189]. **g** Fully hardware-implemented memristor convolutional neural network based on array-level computation [189]. Reproduced with permission. Copyright 2020, Springer Nature

Biological neural networks have inspired a broad range of artificial neural network models, which now serve as the computational backbone of artificial intelligence. From a task perspective, neural network-inspired memristive systems can be broadly related to two directions: AI acceleration for high-throughput computation and neuromorphic computing for biologically inspired, event-driven information processing [40–42]. Different neural network paradigms impose different requirements on the underlying hardware, and memristors may provide distinct advantages depending on the target computational scenario, such as temporal information processing, event-driven sensing, or image recognition. Representative examples include reservoir computing (RC), spiking neural networks (SNNs), and convolutional neural networks (CNNs), which are among the most widely discussed network types in neuromorphic hardware research. In addition, array-level platforms such as 1T1M crossbar chips integrated with peripheral circuits provide a practical route toward implementing these network models in memristive hardware, as illustrated in Fig. 3a [43–45].

Reservoir computing is particularly suitable for temporal and time series signal processing because it can efficiently handle dynamic, time-varying inputs [46]. In a typical RC framework, the input is first preprocessed through time multiplexing and mask matrix multiplication, then converted into a voltage pulse sequence, and applied to a memristive reservoir. The dynamic responses of virtual nodes are sampled and organized as reservoir states, and the final output is obtained through a trained linear readout, as shown in Fig. 3b, c. Owing to their dynamic conductance evolution and temporal response capability, memristors are especially attractive for this type of computing architecture [47–49].

Spiking neural networks are more suitable for event-driven and sparse information processing because they treat spikes as the fundamental carriers of information and explicitly preserve temporal relationships between neurons. A typical SNN consists of input, hidden, and output layers in which information is transmitted in the form of discrete spike trains and processed through spike-dependent synaptic weight modulation, as shown in Fig. 3d [50–52]. This event-driven computation paradigm can effectively reduce redundancy and energy consumption when processing dynamic sensory inputs [53]. In a representative case, a dynamic vision sensor converts real scene changes into event streams, which are then processed by event-driven hardware, as shown in Fig. 3e. In this context, memristors are especially promising because their pulse-driven conductance modulation and synaptic plasticity naturally resemble the temporal and event-based behavior of biological synapses [54–57].

Convolutional neural networks are widely used for image recognition, feature extraction, and other vision-related tasks because they efficiently process spatially structured data through hierarchical convolution operations [58, 59]. A typical CNN includes input, convolutional, pooling, fully connected, and output layers, as shown in Fig. 3f. Since CNNs require large-scale multiply–accumulate operations, memristor crossbar arrays are especially attractive for accelerating these computations in an in-memory manner [60–62]. As illustrated in Fig. 3g, convolution kernels can be mapped onto memristor arrays, thereby improving parallelism and reducing data movement overhead. This makes memristive hardware particularly relevant to CNN-based artificial intelligence tasks [63–66].

Overall, different neural network paradigms correspond to different task demands, and the advantages of memristors should therefore be understood in a task-oriented way. RC is closely related to temporal signal processing, SNN is highly compatible with event-driven and low-power computation, and CNN is well suited for image processing tasks that benefit from large-scale parallel in-memory operations. Accordingly, temporal signal tasks place greater emphasis on dynamic conductance evolution and short-term memory, event-driven tasks rely more strongly on spike-dependent plasticity and low-power operation, whereas image processing tasks require high-parallelism multiply–accumulate capability and array-level integration [67]. This task-oriented perspective helps clarify why memristors are increasingly regarded as promising hardware platforms for neuromorphic and artificial intelligence computing. This task-based mapping also provides a useful bridge to the following sections, where representative memristor materials, device architectures, performance metrics, and application scenarios are discussed in a more hardware-oriented framework [68–70].

## Memristor

**Fig. 4:**
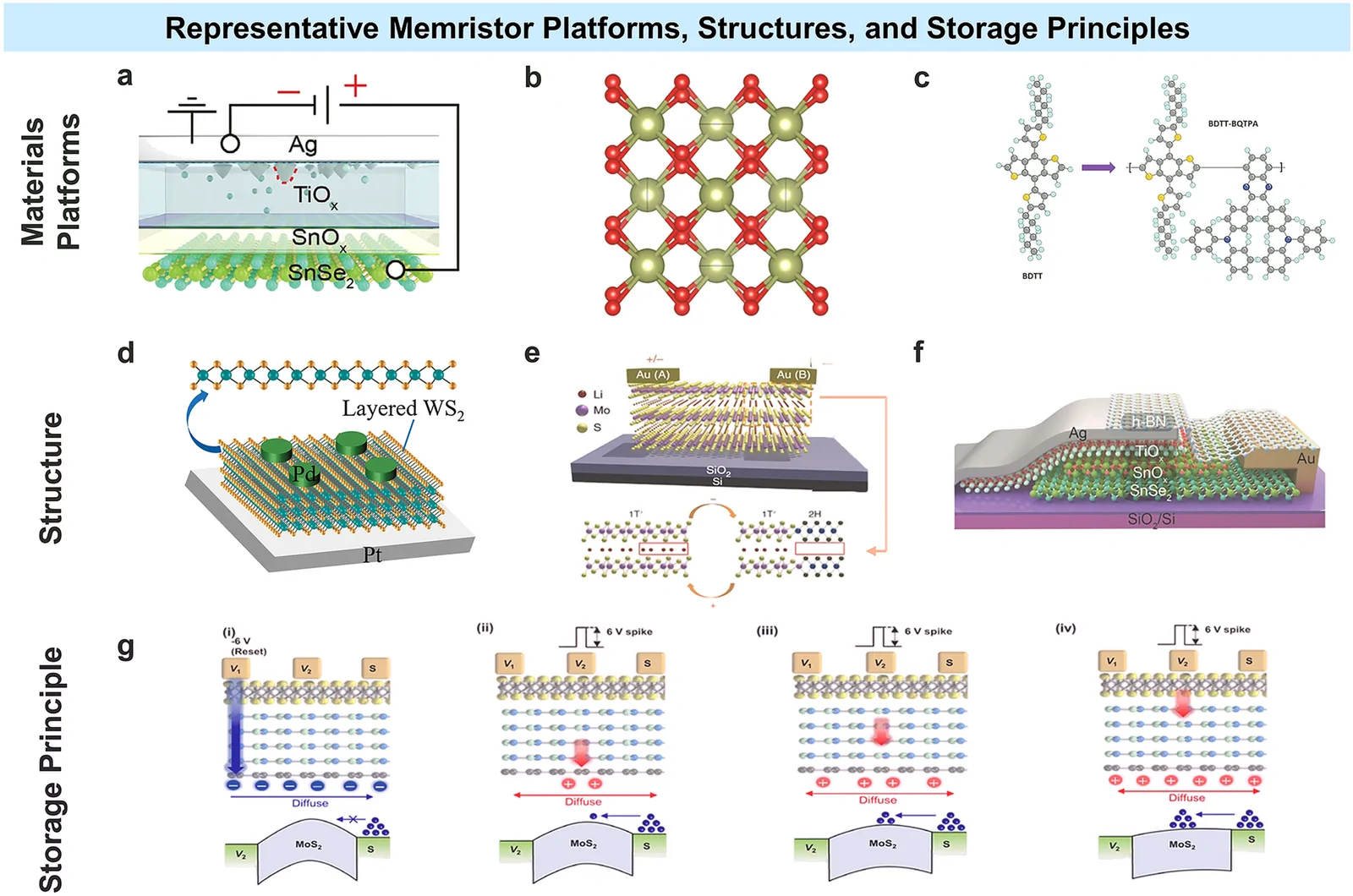
Representative memristor platforms, structures, and storage principles relevant to neuromorphic computing. **a** Representative vertical oxide/heterostructure memristor illustrating cross-sectional device architecture and interfacial transport pathway [190]. Reproduced with permission. Copyright 2024, John Wiley and Sons. **b** Representative crystal structure related to HfO₂-based ferroelectric systems [191]. Reproduced with permission. Copyright 2024, Springer Nature. **c** Representative polymer material platform for nanomemristors [192]. Reproduced with permission. Copyright 2021, Springer Nature. **d** WS₂-based layered memristor structure for low-power neuromorphic computing [193]. Reproduced with permission. Copyright 2019, John Wiley and Sons. **e** Planar MoS₂ memristor with ionic modulation and phase transition-related switching behavior [34]. Reproduced with permission. Copyright 2019, Springer Nature. **f** Integrated heterostructure/selector-assisted memristor architecture for low-power memory and practical circuit applications [190]. Reproduced with permission. Copyright 2024, John Wiley and Sons. **g** Multiterminal floating-gate memristor illustrating charge-modulated storage and multilevel neuromorphic functionality [186]. Reproduced with permission. Copyright 2023, Springer Nature

The memristor was first proposed by Leon Chua in 1971 as the fourth fundamental circuit element, complementing the resistor, capacitor, and inductor by establishing a direct relationship between electric charge and magnetic flux linkage [71, 72]. This concept filled the missing link among the four fundamental circuit variables: current, charge, voltage, and flux linkage. In 2008, HP Labs experimentally demonstrated the memristive effect in a TiO_2_/TiO_2-x_ structure, thereby validating Chua’s theoretical prediction and drawing widespread attention to memristors as promising hardware platforms for non-von Neumann computing. Since then, memristor research has developed rapidly from conceptual verification to material diversification, structural innovation, and application-oriented system design. To provide readers with a clearer overview of this evolution, this section first reviews the development history of memristors and then summarizes representative material systems, device structures, and storage principles, as illustrated in Fig. 4, before discussing performance optimization [73–77].

### Development History

The development of memristors can be more clearly understood as a progressive evolution from device-level synaptic emulation to system-level neuromorphic and artificial intelligence-oriented hardware [78]. After the memristor concept was experimentally validated, early studies mainly focused on demonstrating whether a single memristive device could reproduce key biological synaptic functions. Representative works showed that memristors could emulate habituation/fatigue behavior, short-term memory to long-term memory transition, and other basic synaptic responses, thereby establishing their feasibility as artificial synapses [79–81]. Subsequently, diffusive memristors further improved the biomimetic fidelity of memristive devices by reproducing temporal dynamics analogous to biological Ca^2+^-regulated plasticity, while crossbar-based memristor networks enabled sparse coding and efficient sensory data representation [82, 83]. These studies marked the transition of memristor research from simple resistive switching devices to functional neuromorphic elements capable of synaptic emulation and bio-inspired information encoding.

A second major stage in memristor development was the move from single-device demonstrations to trainable neural network hardware. In this stage, the emphasis shifted toward array-level implementation, in situ learning, and integration with peripheral circuits. Efficient and self-adaptive in situ learning in multilayer memristor neural networks demonstrated that memristor crossbars could be directly used for online training while tolerating hardware imperfections, representing an important step toward practical artificial intelligence hardware [84, 85]. This was followed by the development of three-dimensional monolithically integrated hardware neural systems, which showed that volatile and nonvolatile memristive devices could be vertically integrated with CMOS circuits to realize on-chip neuromorphic processing [83, 86]. At the same time, device engineering strategies such as alloying conducting channels were introduced to improve switching uniformity, programmed symmetry, and multilevel retention, addressing one of the major bottlenecks in analog memristor computing [87, 88]. These advances indicate that the historical development of memristors has been driven not only by new switching media, but also by the urgent need for large-array reliability, training capability, and compatibility with hardware neural systems [89].

More recent studies have expanded the scope of memristor research from neural network training to diverse computing paradigms and multifunctional intelligent systems. For example, memristive control of mutual spin Hall nano-oscillator synchronization demonstrated that memristors can serve as nonvolatile control elements in oscillator-based neuromorphic hardware, extending their role beyond conventional resistive memory and synaptic weighting [90, 91]. Optoelectronic and visual functionalities have also become increasingly important. Multispectral color-cognitive memristors showed that two-terminal memristors can be endowed with wavelength-dependent synaptic behavior for neuromorphic visual systems, while flexible memristors with enhanced linearity and precise weight tuning further highlighted the importance of mechanical adaptability and analog update symmetry in practical neuromorphic computing [92, 93]. In parallel, cellular-automata-embedded memristor-based recirculated logic demonstrated that memristors can support low-cost logic-in-memory architectures beyond standard neural network tasks, indicating a broader trend toward algorithm-specific in-memory computing [94, 95]. These studies suggest that memristor development has progressively expanded from conventional synaptic emulation to vision, logic, oscillatory computing, and task-specific hardware acceleration [96].

**Fig. 5:**
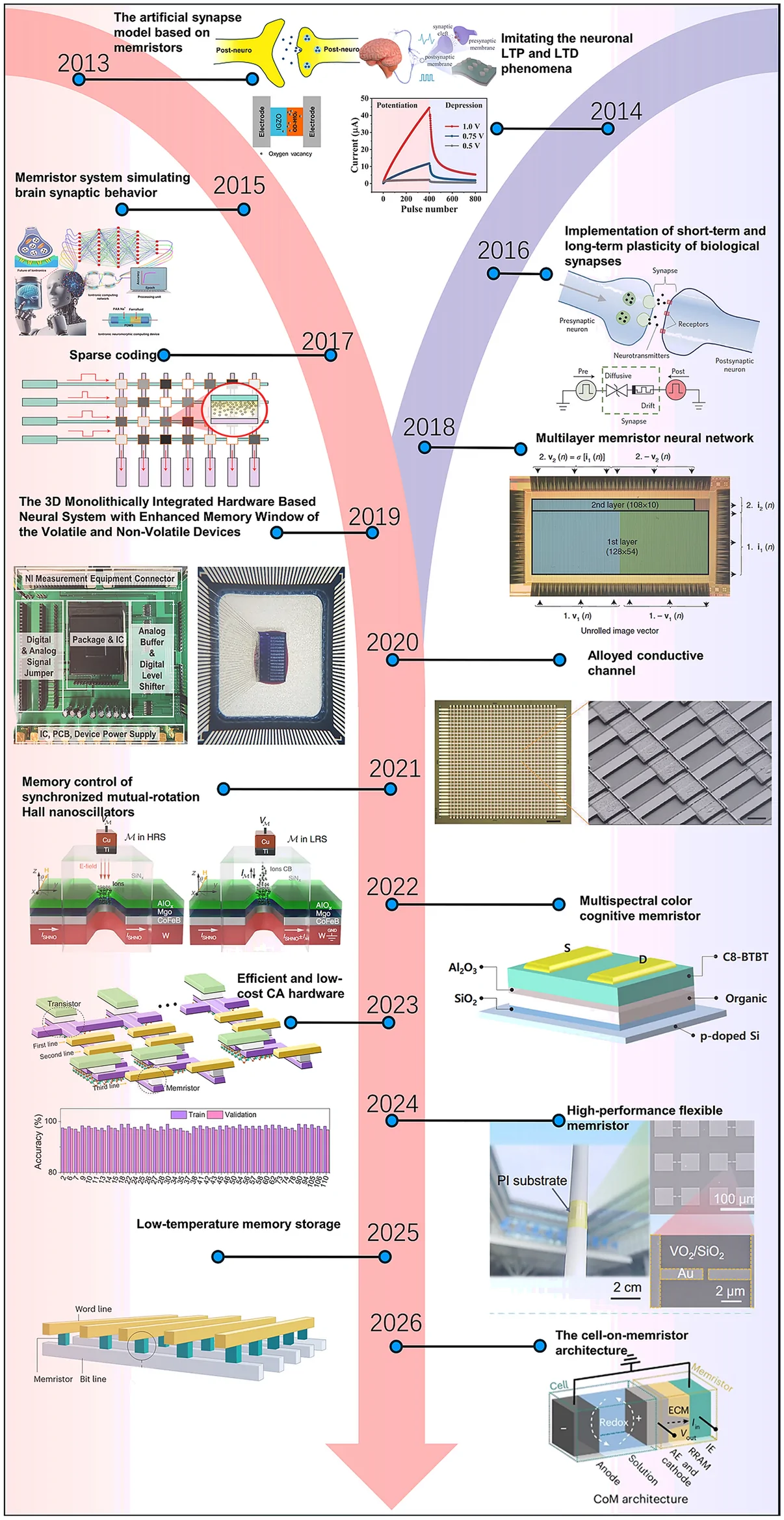
Development timeline of neuromorphic memristors from 2013 to 2026. Representative milestones along the timeline illustrate the evolution of the field from device-level synaptic emulation, to trainable neural network hardware, to multifunctional intelligent systems, and finally to flexible, multimodal, and unconventional computing platforms. Reproduced with permission. Copyright 2015-2018, 2020-2026 Springer Nature. Reproduced with permission. Copyright 2019, John Wiley and Sons

In the latest stage of development, memristors are increasingly being explored in flexible, crossmodal, self-powered, and extreme environment systems. Crossmodal sensory neurons based on flexible memristors have shown that memristive devices can directly fuse multimodal signals such as pressure and temperature and enable real-time in-sensor computing for wearable human–machine interfaces. At the same time, iontronic fluidic memristive and memcapacitive devices have opened a route toward self-powered neuromorphic electronics with strong bio-environment compatibility [97–99]. More recently, the emergence of cryogenic memristors has extended memristor research into low-temperature unconventional computing, suggesting potential relevance for future quantum-compatible or cryogenic information processing platforms. Therefore, the development history of memristors should no longer be viewed simply as the optimization of resistive switching characteristics. Instead, it reflects a broader evolution toward reliable, trainable, flexible, multimodal, and application-specific hardware platforms for artificial intelligence, neuromorphic sensing, and unconventional computing. The stage-based development trajectory discussed above is schematically summarized in Fig. 5 [93–95].

### Representative Memristor Platforms, Structures, and Storage Principles

The rapid development of neuromorphic memristors has been closely associated with the diversification of representative platforms, device architectures, and storage mechanisms. Rather than being limited to a single switching medium or structural form, contemporary memristor research has gradually evolved toward a broad design space that includes oxide-based vertical or heterostructure platforms, ferroelectric oxides, polymers, layered van der Waals structures, planar ion-coupled devices, and integrated selector memory architectures. As summarized in Fig. 4, the performance and functionality of a memristor are determined not only by the constituent materials themselves, but also by how the device platform and structure regulate ion migration, polarization evolution, defect formation, interfacial transport, and charge storage [100–103].

From the perspective of representative platforms, oxide-based vertical or heterostructure memristors have become increasingly important because they combine compact device geometry with strong interfacial tunability, making them attractive for low-power memory and neuromorphic functionalities, as illustrated in Fig. 4a. In parallel, HfO₂-based ferroelectric systems remain another major platform because of their CMOS compatibility, rich phase-dependent electrical behavior, and strong potential for reliable switching and dense integration, as represented by the crystal structure in Fig. 4b. In addition, polymer memristors constitute a distinct branch for lightweight, low-power, and mechanically compliant electronics, owing to their structural deformability and potential for homogeneous switching in large-area or nanoscale devices, as shown in Fig. 4c. Together, Fig. 4a–c indicates that modern memristor research is no longer organized only around isolated material classes, but increasingly around representative platforms that combine materials, interfaces, and device geometry [104–106].

In terms of device architecture, vertical, layered, planar, and integrated structures provide different routes toward neuromorphic functionality. The representative vertical oxide/heterostructure platform in Fig. 4a highlights the importance of compact out-of-plane transport and interfacial modulation in practical memristor design. Layered two-dimensional memristors such as the WS₂-based structure in Fig. 4d benefit from well-defined van der Waals interfaces and defect-controlled transport, which are favorable for low-power neuromorphic operation [107–109]. Planar ion-coupled devices based on MoS₂, as shown in Fig. 4e, further demonstrate that in-plane ionic migration can induce local phase transitions and memristive switching, enabling synaptic cooperation and competition behaviors that are difficult to realize in conventional filamentary devices. In contrast, integrated or selector-assisted architectures, presented in Fig. 4f, emphasize controllable switching, reduced sneak-path current, and compatibility with practical memory or in-memory computing circuits. These examples indicate that memristor design has shifted from simple two-terminal switching units toward more application-oriented architectures that simultaneously consider switching controllability, energy efficiency, and system integration [110–113].

Beyond material platforms and structures, the storage principle of memristors is equally important for understanding their neuromorphic functions. Traditional memristive switching is often associated with the formation and rupture of conductive filaments, typically involving oxygen vacancies or active metal ions. However, more recent studies have revealed a broader set of storage mechanisms, including interfacial transport modulation, ferro-ionic motion, defect-mediated hopping, phase transition-assisted switching, and charge storage in floating-gate or multilevel structures. For instance, the planar MoS₂ system in Fig. 4e illustrates how ionic modulation and local phase transition can produce memristive behavior and more complex spatiotemporal signal interactions than those available in conventional vertical switching devices. Likewise, the multiterminal floating-gate memristor shown in Fig. 4g demonstrates that charge modulation in a shared floating gate can realize multineuron connection and more complex neuromorphic operations than those available in conventional two-terminal devices [114, 115]. These developments show that the storage principle of a memristor should not be viewed merely as the physical origin of resistance change, but rather as the foundation that determines whether the device is better suited for nonvolatile weight storage, volatile threshold dynamics, multichannel signal processing, or more advanced neuron–synapse co-functionality [116, 117].

Overall, the representative systems shown in Fig. 4 illustrate that modern memristor research is no longer focused only on achieving resistive switching itself. Instead, it increasingly emphasizes the coordinated design of representative platforms, structures, and storage principles to meet the requirements of specific neuromorphic tasks. Oxide-based and vertical heterostructure platforms are attractive for compact integration and interfacial switching control, ferroelectric oxides are promising for polarization-based switching and CMOS-compatible integration, polymer systems are suitable for lightweight and low-power applications, while layered, planar, and advanced multiterminal architectures enable ion-coupled dynamics, multilevel memory, selector functionality, and biologically inspired information processing. Therefore, a comprehensive discussion of memristors should consider these three aspects together, because only their combined optimization can support the further development of high-performance neuromorphic hardware [118, 119].

## Performance Optimization and Functional Engineering

**Fig. 6:**
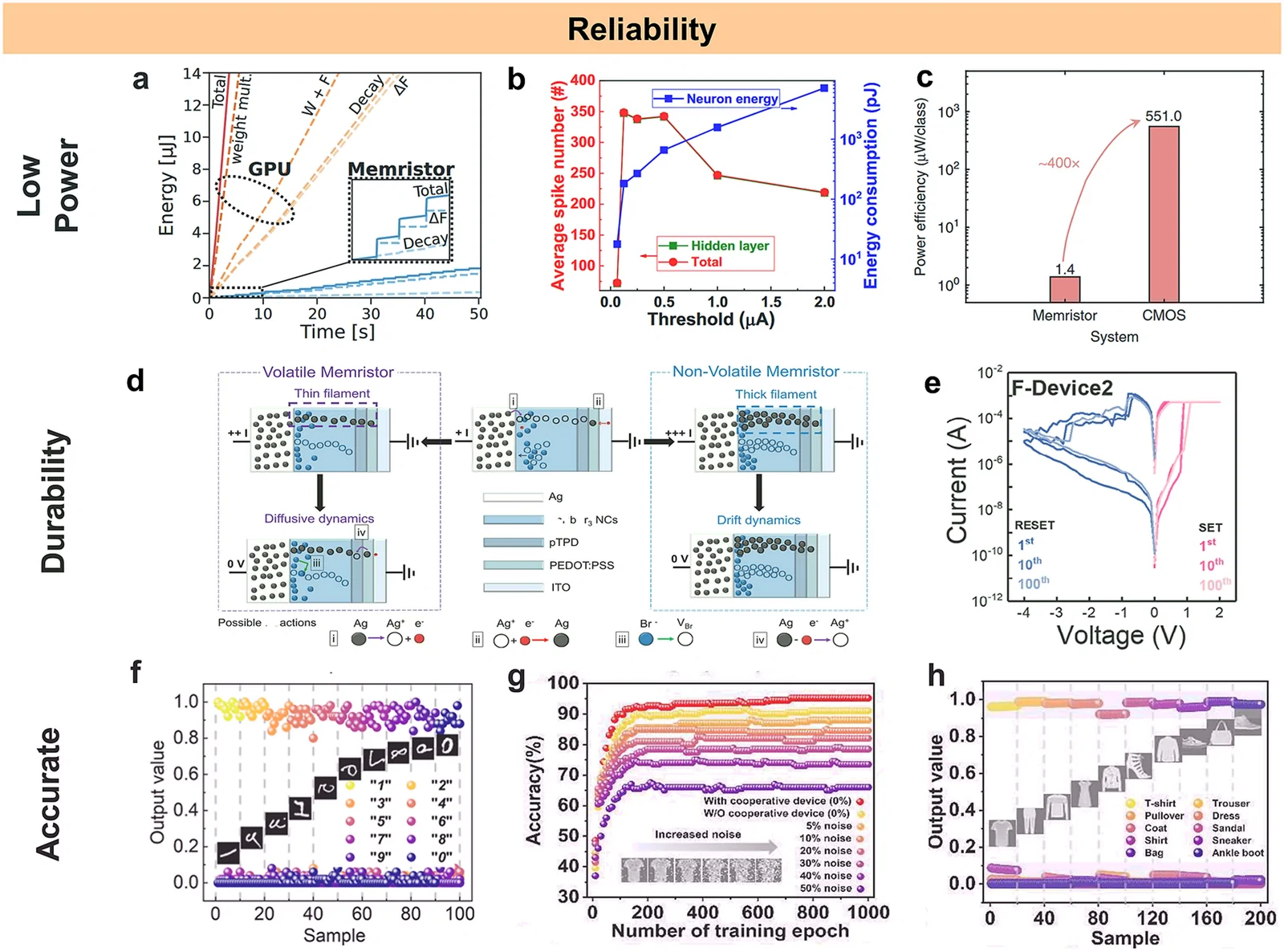
Representative strategies for performance optimization of neuromorphic memristors. **a** Energy comparison between GPU-based computation and multifunctional memristive synapses [194]. Reproduced with permission. Copyright 2024, Springer Nature. **b** Threshold-dependent spike activity and neuron energy consumption of an antiferroelectric transistor neuron [22]. Reproduced with permission. Copyright 2022, Springer Nature. **c** Power efficiency comparison between memristor-based and CMOS-based neural signal analysis systems [2]. Reproduced with permission. Copyright 2020, Springer Nature. **d** Reconfigurable volatile/nonvolatile switching modes in a halide perovskite memristor [195]. Reproduced with permission. Copyright 2022, Springer Nature. **e** Stable repeated switching characteristics of the reconfigurable memristor device [14]. Reproduced with permission. Copyright 2025, John Wiley and Sons. **f** Output distribution for digit recognition based on an antiferroelectric synapse/neuron device [128]. Reproduced with permission. Copyright 2024, Springer Nature. **g** Recognition accuracy under different noise levels in cooperative dendritic memristor-based learning [36]. Reproduced with permission. Copyright 2024, Springer Nature. **h** Output distribution for fashion pattern recognition enabled by cooperative dendritic memristive devices [36]. Reproduced with permission. Copyright 2024, Springer Nature

**Fig. 7:**
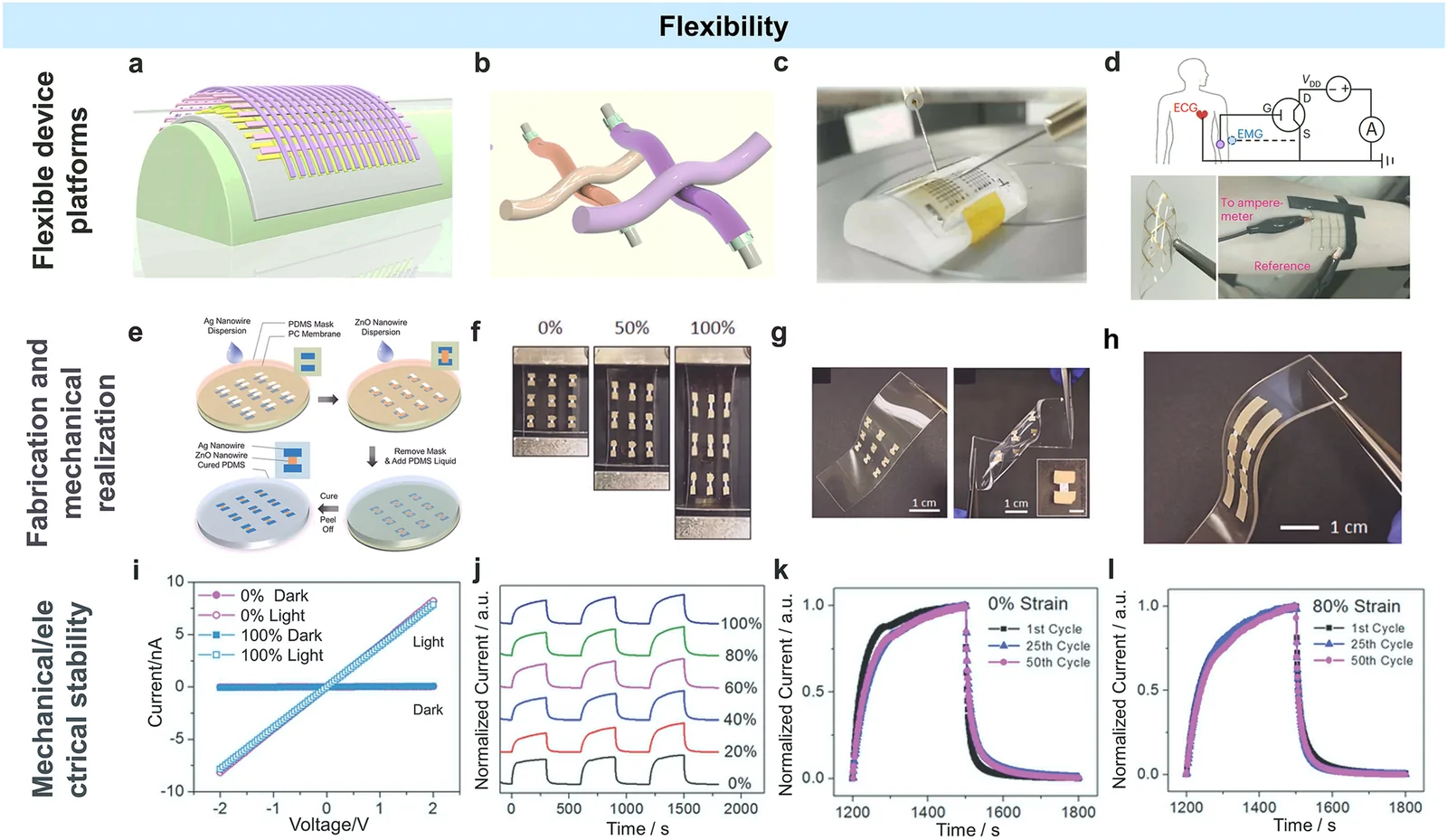
Representative flexible and wearable neuromorphic platforms, fabrication strategies, and mechanical/electrical stability. **a** Flexible 3D memristor array [119]. Reproduced with permission. Copyright 2021, John Wiley and Sons. **b** Reconfigurable textile memristor network [62]. Reproduced with permission. Copyright 2022, Springer Nature. **c** Flexible organic electrochemical transistor device [119]. Reproduced with permission. Copyright 2021, John Wiley and Sons. **d** Wearable multimodal sensing and on-skin integration [61]. Reproduced with permission. Copyright 2023, Springer Nature. **e** Lithographic filtration process for stretchable nanowire device fabrication [149]. Reproduced with permission. Copyright 2014, John Wiley and Sons. **f** and** h** Fabrication process and device demonstrations of an intrinsically stretchable nanowire photodetector. Reproduced with permission. Copyright 2014, John Wiley and Sons. **i** and** j**; **k** and** l** Electrical and photoresponse stability of the stretchable device under different strain levels and repeated cycles [149]. Reproduced with permission. Copyright 2014, John Wiley and Sons

Recent progress in neuromorphic memristors shows that performance optimization is no longer restricted to conventional switching metrics alone. Instead, current studies increasingly focus on whether memristive devices can simultaneously satisfy multiple application-oriented requirements, including low power consumption, operational durability, recognition accuracy, mechanical flexibility, and wearable integration. Accordingly, the discussion in this section is organized around two complementary level [120–122]. Figure 6 summarizes representative strategies for improving low-power operation, durability, and accuracy, whereas Fig. 7 focuses on flexibility, mechanical robustness, and wearable integration. This organization helps clarify how different optimization targets support different forms of neuromorphic hardware [123, 124].

### Function-Oriented Device Optimization

One of the most important recent trends in neuromorphic memristor research is that device optimization is becoming increasingly function-oriented [125, 126]. In earlier studies, performance improvement mainly focused on conventional electrical parameters such as switching ratio, retention, and endurance. However, as neuromorphic computing tasks have become more complex, researchers have begun to emphasize whether a memristive device can directly support richer biological and computational functions, including multifunctional synaptic dynamics, compact neuron implementation, reconfigurable volatile/nonvolatile operation, mechanical flexibility, multichannel signal processing, and efficient analog system integration [127]. As summarized in Fig. 6, recent optimization strategies are therefore no longer limited to improving resistive switching itself, but are increasingly directed toward enhancing the target neuromorphic function of the device [128–130].

In addition to energy consumption, endurance, and recognition accuracy, programming time is another critical metric for practical neuromorphic memristors. Depending on the device mechanism, material system, and biasing scheme, programming latency may range from nanoseconds to milliseconds, which directly affects training throughput, online adaptation speed, and overall system latency [131–133]. Therefore, programming time should be considered together with switching energy and reliability when evaluating the practical applicability of memristive hardware for artificial intelligence-oriented tasks.

A representative example is shown in Fig. 6a, where a single SrTiO_3_-based neuromorphic memristor closely emulates multiple synaptic mechanisms within one device. Unlike conventional memristors that mainly provide long-term weight storage, this device can simultaneously reproduce long-term memory, short-term memory, short-term plasticity, and meta-plasticity [134]. Such multifunctionality is highly valuable for bio-inspired neural networks that require more realistic synaptic dynamics. Importantly, the device operates in a non-filamentary and low-conductance regime, which is beneficial for stable and energy-efficient operation, and the corresponding neural network implementation shows about two orders of magnitude lower energy consumption compared with a pure GPU-based solution. This example indicates that enriching intrinsic synaptic functionality is itself becoming a major optimization target [135, 136].

Another important optimization strategy is to improve compactness and reduce peripheral hardware overhead in artificial neuron or synapse implementation. As shown in Fig. 6b, the antiferroelectric transistor neuron based on Hf_0.2_Zr_0.8_O_2_ uses intrinsic polarization/depolarization processes to realize leaky integrate-and-fire behavior without external capacitors or reset circuits. This greatly simplifies the neuron architecture while maintaining low energy consumption, high endurance, and good uniformity [131, 137]. Similarly, the two-terminal antiferroelectric HZO device shown in Fig. 6f demonstrates that artificial neuron-like volatile behavior and synaptic plasticity can be achieved in closely related device platforms through work function engineering. In that system, W/HZO/W devices realize leaky integrate-and-fire characteristics, whereas Au/Ti/HZO/W devices exhibit synaptic functions such as PPF and LTP/LTD, enabling digit recognition accuracy above 90% [138–140]. These studies show that reducing circuit complexity while preserving neuron/synapse functionality is another key direction in performance optimization.

In addition to compactness, reconfigurability has become an increasingly important optimization objective. The reconfigurable halide perovskite nanocrystal memristor shown in Fig. 6d provides a good example [141–143]. By controlling electrochemical reactions, the same device platform can switch on demand between volatile diffusive mode and nonvolatile drift mode, allowing it to meet different hardware requirements in reservoir computing, spiking neural networks, and artificial neural networks. This capability is particularly important because different neuromorphic frameworks often demand different timescales and memory characteristics. Therefore, the optimization of memristors is moving beyond single-mode operation toward multifunctional and reconfigurable hardware substrates that can support multiple computational primitives within one platform [144].

Mechanical adaptability and biologically realistic signal processing have also become important optimization targets. As shown in Fig. 6e, flexible synaptic memristors based on zirconium-oxo clusters improve device reliability and analog conductance symmetry by controlling the rigidity of the switching matrix, thereby achieving both stable bending performance and high-precision neuromorphic computing. In parallel, the ionic diffusive dendritic nanomemristor shown in Fig. 6g realizes multichannel many-to-one modulation, dendritic competition/cooperation, and ultra-low-voltage operation down to 80 mV, which is close to the biological action potential level. These examples indicate that optimization is no longer confined to conventional static memory characteristics, but is increasingly extended toward flexible, wearable, and spatiotemporal neuromorphic signal processing scenarios [145, 146].

Finally, the optimization of memristors should also be judged from a system-level perspective. The memristor array-based neural signal analysis system shown in Fig. 6c demonstrates that memristors can directly process analog neural signals with high efficiency in brain–machine interface-related tasks. In that work, memristor arrays were used to implement filtering and identification of epilepsy-related neural signals, achieving an accuracy of 93.46% and nearly 400-fold improvement in power efficiency compared with state-of-the-art CMOS systems. This result suggests that the most meaningful device optimization is not only to improve single-device parameters, but also to enhance the practical efficiency, compactness, and scalability of complete neuromorphic information processing systems [118, 119].

Overall, the representative examples in Fig. 6 show that performance optimization of neuromorphic memristors is increasingly application-driven and function-oriented. The most competitive devices are no longer those that merely exhibit good resistive switching characteristics, but those that can simultaneously provide multifunctionality, compactness, reconfigurability, flexibility, biological plausibility, and system-level efficiency. Therefore, future optimization strategies should focus on the coordinated design of material properties, device architecture, and target computational function.

### Flexibility, Mechanical Robustness, and System-Level Wearable Integration

For neuromorphic devices targeting wearable, bio-integrated, and edge computing applications, performance optimization should also be evaluated from the perspective of flexibility, mechanical robustness, and system-level integration. In these scenarios, a device is not only required to exhibit stable electrical switching, but also to maintain its memory and neuromorphic functions under bending, stretching, interwoven assembly, or direct attachment to curved surfaces. Therefore, flexibility should be regarded as a core performance metric for practical neuromorphic hardware rather than merely a packaging-related property, as reflected by the representative flexible platforms, fabrication strategies, and stability evaluations summarized in Fig. 7.

A first important route is the development of flexible neuromorphic device platforms with preserved storage and computing capability. The flexible 3D memristor array shown in (Fig. 7a) demonstrates that high-density neuromorphic hardware can be constructed on flexible substrates through low-temperature ALD, while still retaining multibit storage and synaptic plasticity. The three-layer crossbar structure, low-temperature fabrication process, and stable switching behavior in different layers together indicate that flexible integration and high-density architecture can be realized simultaneously. Similarly, the reconfigurable textile memristor network in Fig. 7b shows that fiber-shaped architectures can support both synaptic and neuronal functions within an interwoven wearable format, which is particularly attractive for smart textile electronics [147, 148]. Together, Fig. 7a, b indicates that flexibility optimization is closely related to device architecture and fabrication compatibility, especially when high-density integration or woven system assembly is required [149].

A second route is to integrate sensing, memory, and processing into flexible homogeneous platforms. The organic electrochemical transistor shown in Fig. 7c provides a representative example, because its vertical traverse architecture enables dual operation as a volatile receptor and a nonvolatile synapse. This type of design is highly valuable for flexible neuromorphic systems, since it reduces device heterogeneity and facilitates monolithic integration of sensing and computation. The wearable demonstration in Fig. 7d further highlights that such flexible platforms are not limited to isolated device operation, but can be directly adapted to on-skin or bio-interface scenarios, where multimodal signals must be acquired and processed in a mechanically compliant form [150–152].

Besides functional platform design, fabrication strategy is also crucial for achieving mechanically robust flexible electronics. The lithographic filtration process illustrated in Fig. 7e shows how different nanowire layers can be sequentially patterned and embedded into an elastomer matrix, thereby forming intrinsically stretchable device structures [153]. The corresponding device demonstrations in Fig. 7f–h further show that such embedded architectures can maintain structural integrity under tensile deformation, array-level realization, and conformal attachment. Although this nanowire photodetector is not itself a memristor, the fully embedded structural concept presented in Fig. 7e–h provides useful design guidance for future stretchable neuromorphic systems that aim to integrate sensing and signal processing in soft or skin-like electronics [154].

The importance of flexibility optimization is ultimately determined by whether the electrical behavior remains stable under deformation. The current–voltage characteristics in Fig. 7i indicate that the device can preserve clear photoresponse behavior under both unstrained and highly strained states. The dynamic responses under different strain levels shown in Fig. 7j further suggest that mechanical deformation mainly modifies the response magnitude and kinetics, while the overall sensing function remains preserved. More importantly, the cycling results at 0% strain and high strain in Fig. 7k, l show that repeated operation does not lead to obvious degradation of the normalized response, demonstrating that electrical stability and mechanical compliance can be maintained simultaneously. This type of stability evaluation is essential for flexible neuromorphic hardware, because practical wearable systems must operate reliably not only in static states but also during repeated deformation and long-term use.

In addition to maintaining stable operation under deformation, flexible neuromorphic devices should also be evaluated in terms of system-level integration. Flexible 3D arrays such as Fig. 7a, textile-based reconfigurable memristor networks such as Fig. 7b, and multimodal sensing–memory–processing platforms such as Fig. 7c, d indicate that flexibility optimization is meaningful only when it can support complete wearable neuromorphic systems rather than isolated device demonstrations. In this sense, the optimization of flexible neuromorphic hardware should include not only mechanical endurance and electrical stability, but also functional matching among storage, sensing, and computation units at the system level [155].

Overall, the examples in Fig. 7 show that flexibility-related optimization of neuromorphic hardware has evolved far beyond simple bending tests. Flexible high-density arrays (Fig. 7a), textile-based reconfigurable memristor networks (Fig. 7b), multimodal flexible sensing–memory–processing platforms (Fig. 7c, d), embedded stretchable fabrication strategies (Fig. 7e-h), and deformation-tolerant electrical stability (Fig. 7i–l) together indicate that future progress in this area will depend on the coordinated optimization of materials, architecture, mechanics, and system-level application scenarios, rather than on electrical switching performance alone.

## Applications in Artificial Intelligence

**Fig. 8:**
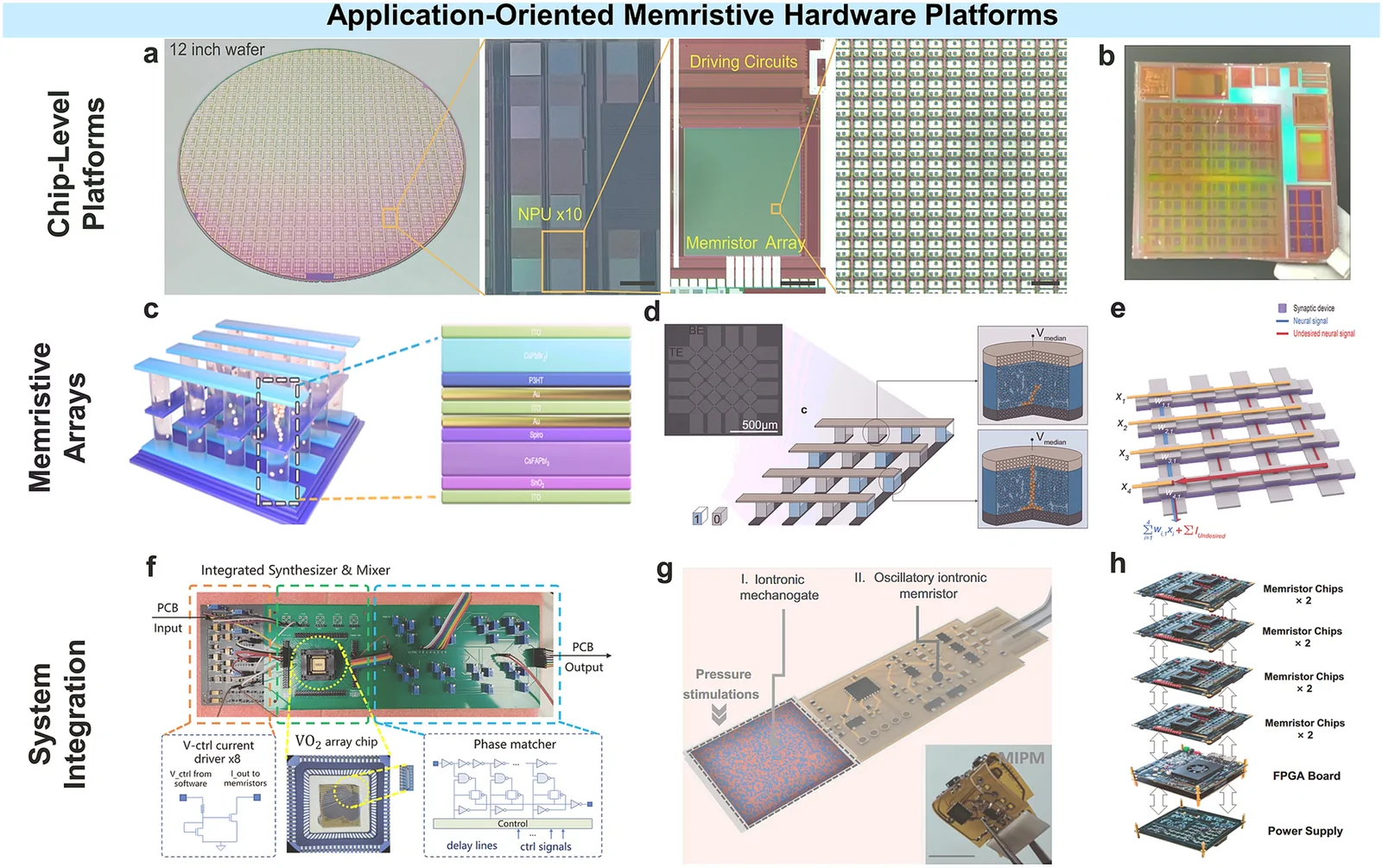
Application-oriented memristive hardware platforms. **a** High-precision memristor chip for analog computing [196]. Reproduced with permission. Copyright 2024, Springer Nature. **b** Hybrid 2D–CMOS microchip for memristive applications [140]. Reproduced with permission. Copyright 2023, Springer Nature. **c** Self-powered memristor array for artificial striate cortex [47]. Reproduced with permission. Copyright 2022, Springer Nature. **d** Tunable stochastic memristor array for encryption and computing [197]. Reproduced with permission. Copyright 2024, Springer Nature. **e** Self-rectifying memristor synaptic array for low-crosstalk neuromorphic systems [15]. Reproduced with permission. Copyright 2018, Springer Nature. **f** VO₂ memristor-based frequency converter system for wireless Internet-of-things applications [168]. Reproduced with permission. Copyright 2024, Springer Nature. **g** Mechano-gated iontronic piezomemristor system for temporal tactile neuromorphic plasticity [198]. Reproduced with permission. Copyright 2025, Springer Nature. **h** Full-stack memristor-based computation-in-memory system with software–hardware co-development [199]. Reproduced with permission. Copyright 2025, Springer Nature

Recent progress in neuromorphic memristors has gradually shifted the focus of the field from isolated device demonstrations to application-oriented hardware systems. In practical artificial intelligence scenarios, the value of memristive devices is no longer determined only by their switching characteristics or synaptic plasticity, but increasingly by whether they can support specific computational functions such as high-precision analog computing, chip-level integration, self-powered visual perception, secure stochastic computing, low-crosstalk synaptic arrays, wireless signal processing, tactile neuromorphic perception, and full-stack computation-in-memory deployment. Therefore, application studies should be organized not as a simple collection of examples, but as a coherent framework linking device characteristics with target system functions. Representative examples are summarized in Fig. 8.

### Application-Oriented Memristive Hardware Platforms

Application studies of neuromorphic memristors are increasingly moving from isolated device demonstrations toward hardware platforms with clear functional roles. As summarized in Fig. 8, these application-oriented platforms can be organized into three levels, namely chip-level platforms Fig. 8a, b, memristive arrays Fig. 8c-e, and system integration Fig. 8f-h. This hierarchical organization is useful because it directly links the hardware form of the memristive platform with its target application in artificial intelligence and intelligent electronics [15, 156].

At the chip level, the main goal is to improve computational precision and hardware compatibility. The high-precision memristor chip in Fig. 8a shows that memristor arrays can support analog computing with arbitrarily high precision through dedicated programming strategies, indicating that memristive hardware can extend beyond low-precision neuromorphic inference toward more accurate analog computing tasks. The hybrid 2D-CMOS microchip in Fig. 8b further demonstrates that memristive devices can be directly integrated with CMOS circuitry to form dense 1T1M cells, enabling in-memory logic operations and synaptic plasticity signals on a practical semiconductor platform. Therefore, Fig. 8a, b together indicates that chip-level applications emphasize precision, controllability, and compatibility with mature microelectronic technology [157, 158].

At the array level, the key issue is whether memristive hardware can support collective perception and learning behaviors in scalable crossbar architectures. The self-powered artificial striate cortex in Fig. 8c shows that memristor crossbar arrays can emulate binocular and orientation-selective receptive fields, thereby enabling more biologically realistic visual information processing [87, 159, 160]. The tunable stochastic memristor array in Fig. 8d indicates that array-level hardware can support both randomness-based security functions and deterministic logic operations under different biases. Meanwhile, the self-rectifying synaptic array in Fig. 8e demonstrates that intrinsic rectification can effectively suppress leakage paths in dense crossbar systems while preserving synaptic functions such as LTP, LTD, and STDP. Taken together, Fig. 8c-e shows that memristive arrays are especially important for applications that require scalable collective computation, perception, and learning [1, 8, 15].

At the system integration level, memristive applications are no longer restricted to conventional neural network acceleration, but are expanding toward more diverse intelligent systems. The VO₂ memristor-based frequency converter in Fig. 8f shows that memristive hardware can be used for in situ frequency synthesis and mixing in wireless Internet-of-things systems, thereby supporting application-specific signal processing tasks. The mechano-gated iontronic piezomemristor in Fig. 8g further extends the application scope toward temporal tactile neuromorphic perception by coupling mechanical sensing with excitatory and inhibitory plastic responses. Finally, the full-stack computation-in-memory system in Fig. 8h demonstrates that the practical deployment of memristive hardware increasingly depends on software–hardware co-development, including compiler support, flexible mapping, and optimization against hardware non-idealities. Thus, Fig. 8f–h together shows that system-level applications require the coordinated integration of devices, arrays, circuits, and algorithms [161].

Overall, Fig. 8 shows that application-oriented memristive hardware platforms can be understood through a clear hierarchy from chips Fig. 8a, b to arrays Fig. 8c-e, to complete systems Fig. 8f-h. This hierarchy also suggests that future progress in neuromorphic memristor applications will depend not only on improving device characteristics, but also on strengthening the correspondence between hardware level and target application scenario.

### Recognition, Perception, and Human–Machine Interaction

**Fig. 9:**
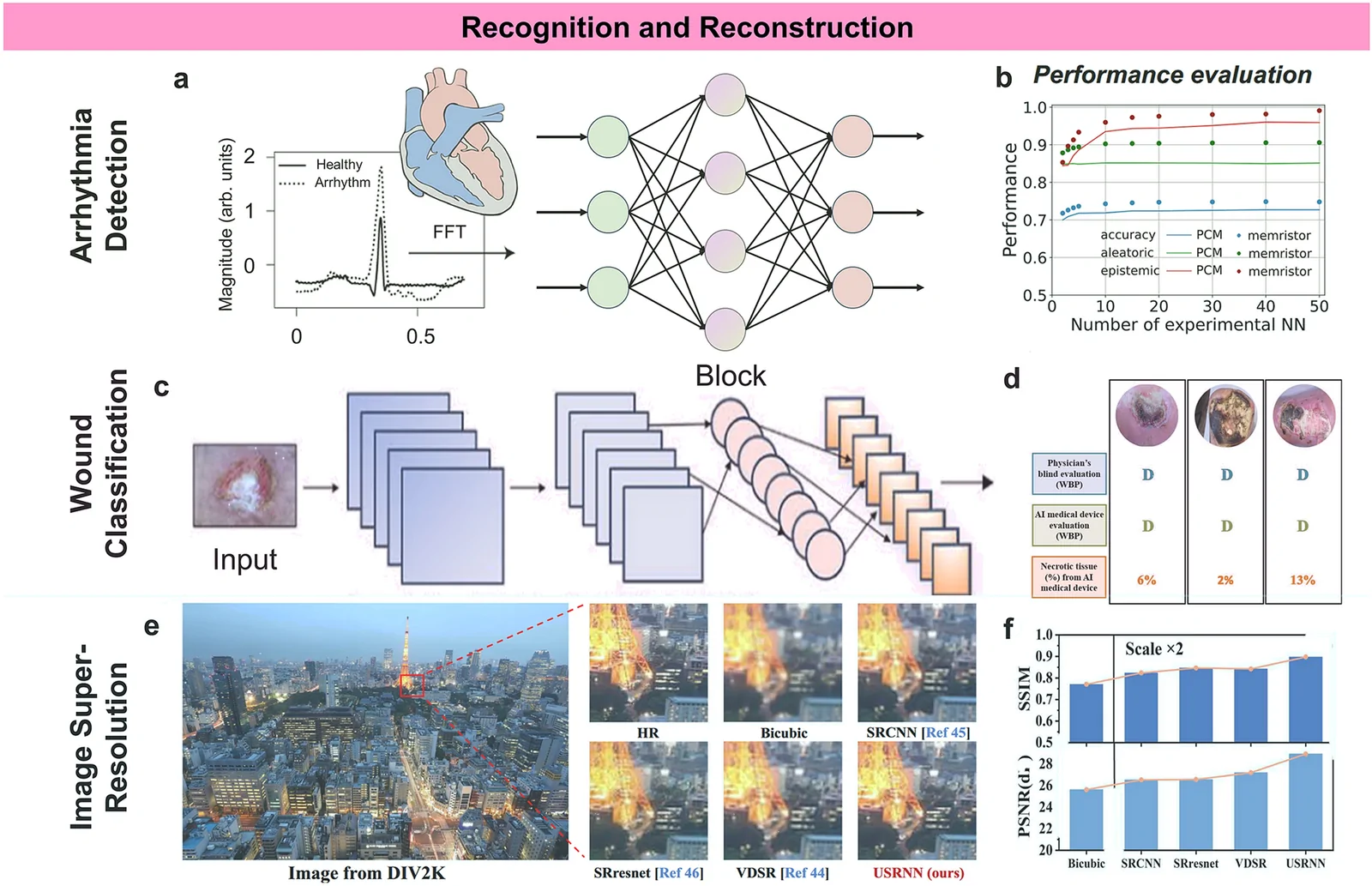
Recognition and reconstruction tasks enabled by memristive neuromorphic hardware. **a** and **b** Memristor-based Bayesian neural network for arrhythmia classification and uncertainty-aware performance evaluation [200]. Reproduced with permission. Copyright 2023, Springer Nature. **c** and **d** Clinically validated chronic wound classification using a memristor-based neural network framework and standardized wound bed evaluation [4]. Reproduced with permission. Copyright 2024, Springer Nature. **e** and **f** Ultra-deep memristive neural network for image super-resolution reconstruction and quantitative performance comparison [23]. Reproduced with permission. Copyright 2024, Springer Nature

**Fig. 10:**
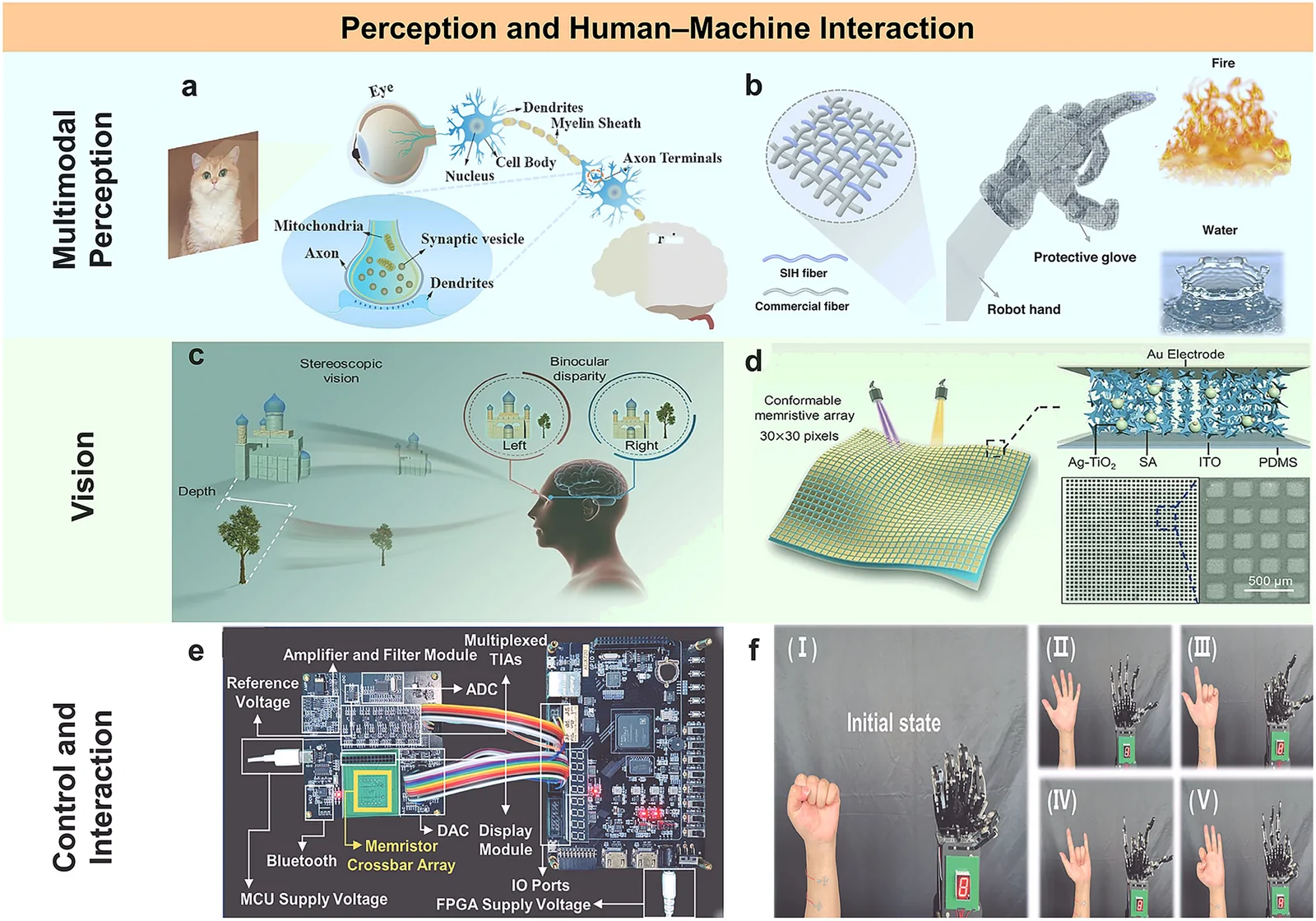
Perception and human–machine interaction enabled by memristive and neuromorphic hardware. **a** Optical–electrical coordinately modulated memristor for artificial vision [201]. Reproduced with permission. Copyright 2024, John Wiley and Sons. **b** Intelligent perceptual textile for danger sensing and touch localization [171]. Reproduced with permission. Copyright 2024, Springer Nature. **c** Hemispherical retina-emulated memristor system for binocular stereo vision [202]. Reproduced with permission. Copyright 2024, John Wiley and Sons. **d** Conformable optoelectronic memristor array for wide-field retinomorphic sensing [202]. Reproduced with permission. Copyright 2024, John Wiley and Sons. **e** FPGA-boosted integrated perception–memory system for physiological signal acquisition and processing [166]. Reproduced with permission. Copyright 2024, John Wiley and Sons. **f** Memristor-assisted human–machine interaction for gesture-based control [166]. Reproduced with permission. Copyright 2024, John Wiley and Sons

Beyond chip-level platforms, memristive applications are increasingly moving toward concrete task demonstrations in recognition, perception, and human–machine interaction. As summarized in Figs. 9 and 10, these task-level studies can be broadly grouped into recognition/reconstruction tasks and perception/control tasks [150, 161]. This organization is useful because it reveals that different applications rely on different hardware priorities, such as confidence-aware classification, deep signal transmission, multimodal sensing, or real-time control [156, 159].

A first important group is recognition-oriented medical and diagnostic tasks, as shown in Fig. 9a-d. The memristor-based Bayesian neural network in Fig. 9a, b demonstrates arrhythmia classification together with uncertainty evaluation, which is especially important for safety–critical edge applications because reliable diagnosis requires not only a prediction, but also an estimate of confidence [162–164]. In parallel, the chronic wound system in Fig. 9c, d uses a two-stage framework in which a conventional CNN first identifies the wound region of interest, whereas the subsequent wound bed analysis and classification are performed by a memristor-based discrete-time cellular neural network. This distinction is important because the CNN is used for ROI detection, while the DT-CNN is responsible for the clinically validated wound classification itself. Taken together, Fig. 9a–d indicates that medical recognition applications place special emphasis on trustworthy inference, standardized decision outputs, and practical validation in realistic scenarios [4].

A second group is reconstruction-oriented vision tasks, presented in Fig. 9e, f. The ultra-deep neural network hardware enabled by light-emitting memristors supports image super-resolution and crosslayer transmission, allowing deep architectures to be extended far beyond the shallow networks typically reported in hardware neural systems [89, 165]. In this case, the application focus is not mainly on uncertainty or classification, but on whether the hardware can sustain deep information flow and preserve reconstruction fidelity, as reflected by metrics such as SSIM and PSNR in Fig. 9f. Therefore, Fig. 9e, f shows that image reconstruction applications require memristive hardware with strong crosslayer transmission capability and high-fidelity deep network support [166, 167].

Another major application direction is multimodal perception, as shown in Fig. 10a, b. The optical–electrical coordinately modulated memristor in Fig. 10a integrates sensing, memory, and processing for artificial vision tasks, showing that visual perception can be partially transferred into the device level rather than separated into sensing and computing modules [2, 92, 168]. The intelligent perceptual textile in Fig. 10b further extends this idea to wearable systems by enabling responses to fire, water, sharp objects, and finger touch. Together, Fig. 10a, b indicates that perception-oriented applications increasingly rely on in-sensor or in-material information processing, where memristive or ion-conductive hardware directly couples external stimuli with memory-like responses [50, 84, 169].

A more specialized perceptual direction is neuromorphic vision, illustrated in Fig. 10c, d. The hemispherical retina in Fig. 10c emulates binocular disparity and stereoscopic vision, while the conformable memristive array in Fig. 10d provides the structural basis for wide field of view, spatial angle detection, and retinomorphic sensing. Compared with the more general perception functions in Fig. 10a, b, the applications in Fig. 10c, d are more strongly oriented toward spatial vision tasks, such as depth perception and motion detection, which require geometry-aware device arrangements in addition to optoelectronic modulation [35, 36, 94]. At the circuit architecture level, retina-inspired visual preprocessing is also closely related to cellular neural network architectures, which provide a local interaction analog framework for spatial filtering, edge enhancement, and retinomorphic image computation [170].

At the system end of the spectrum, control and human–machine interaction are highlighted in Fig. 10e, f. The FPGA-boosted integrated “perception–memory” system in Fig. 10e combines electronic tattoos with memristors to realize physiological signal sensing, recognition, storage, and control in one hardware framework. The robot–hand interaction results in Fig. 10f further show that memristor-assisted systems can support gesture-driven control and real-time human–machine communication. Thus, Fig. 10e, f indicates that interaction-oriented applications require more than sensing alone: they depend on closed-loop integration of perception, memory, decision, and actuation [5, 34].

Overall, Figs. 9 and 10 show that task-level applications of neuromorphic memristors can be understood through a functional progression from recognition and reconstruction to perception and interactive control. Medical recognition tasks (Fig. 9a–d) emphasize confidence and standardized outputs, reconstruction tasks (Fig. 9e, f) emphasize deep information transmission and output fidelity, perception tasks (Fig. 10a-d) emphasize direct coupling between stimuli and memory-processing behavior, and interaction tasks (Fig. 10e, f) emphasize real-time system integration. This comparison suggests that future application studies should not simply accumulate more case demonstrations, but should more clearly match device characteristics with the dominant requirement of each target task.

## Challenges and Outlook

**Fig. 11:**
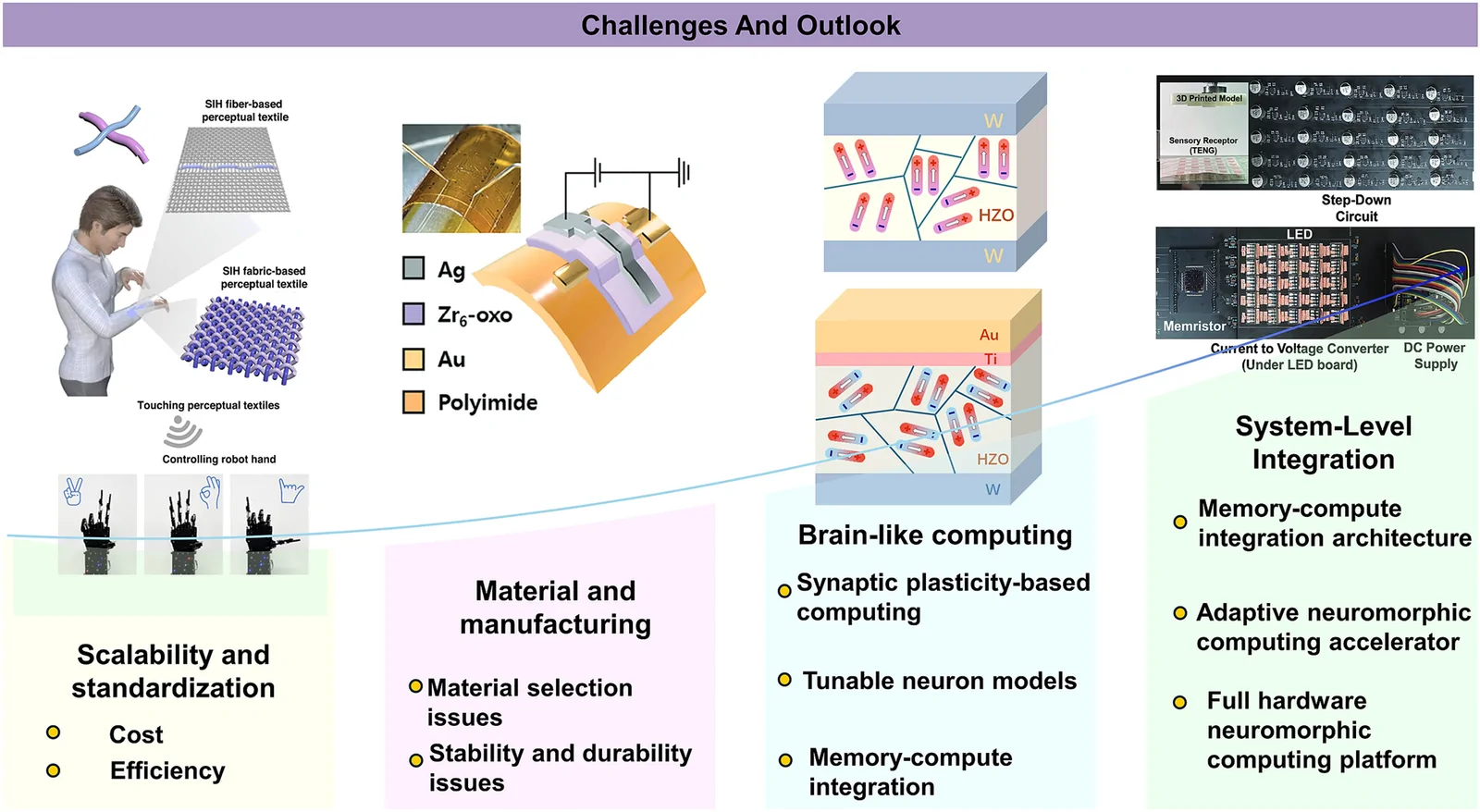
Challenges and outlook for neuromorphic memristor systems. From left to right, the figure summarizes four major directions for future development: scalability and standardization of wearable and perceptual platforms, materials and manufacturing challenges in flexible memristors, the need for more expressive brain-inspired computing architectures, and efficient system-level integration for practical intelligent hardware. Reproduced with permission. Copyright 2024, Springer Nature. Reproduced with permission. Copyright 2025, John Wiley and Sons. Reproduced with permission. Copyright 2025, Springer Nature

Although neuromorphic memristors have made rapid progress in device design, flexible electronics, and application-oriented hardware systems, several key challenges still limit their transition from laboratory demonstrations to practical intelligent platforms. As summarized in Fig. 11, these challenges can be broadly discussed from four perspectives, namely scalability and standardization, materials and manufacturing, brain-inspired computing, and system-level integration. This organization is helpful because it shows that the remaining bottlenecks are no longer confined to device switching itself, but extend to fabrication feasibility, computing architecture, and deployment efficiency [171].

The leftmost panel highlights the challenge of scalability and standardization. Recent perceptual textile studies show that ionically conductive and mechanically robust fibers can be woven into wearable systems capable of danger sensing, touch localization, and human–machine interaction [14]. However, moving from such demonstrations to practical neuromorphic products requires scalable fabrication routes, lower cost, reproducible device behavior, and unified evaluation standards for wearable operation and long-term reliability. Therefore, future progress in this direction depends not only on achieving flexible functionality, but also on establishing standardized methods for fabrication, testing, and application benchmarking [34].

The second panel emphasizes the challenge of materials and manufacturing. Flexible memristors based on zirconium-oxo clusters show that controlled material rigidity can improve repeatability, retention, and analog switching symmetry while maintaining mechanical flexibility [172]. This result makes clear that material selection is not an auxiliary issue, but a central factor that directly determines reliability and neuromorphic precision [128]. At the same time, the dependence on thermally controlled polymerization, optimized switching matrix formation, and stable conductive path evolution also indicates that manufacturing window control remains a major challenge. Thus, future advances will require closer coupling between material design and process engineering.

The third panel points to the challenge of brain-inspired computing. Flexible boron nitride-based memristors have already demonstrated the possibility of integrating in situ digital logic and analog neuromorphic functions within a single system, which suggests a promising route toward more versatile in-memory computing architectures. Nevertheless, current hardware is still far from reproducing the richness and adaptability of biological information processing. In future work, more tunable neuron models, richer synaptic plasticity rules, and tighter memory–compute co-design will be necessary if memristive systems are to move from simple synaptic emulation toward more complete brain-inspired computing platforms.

The rightmost panel stresses the challenge of system-level integration. Recent sensory memory process display systems demonstrate that flexible sensors, memristors, and output modules can be combined to perform tasks such as gait recognition and on-device preprocessing. These studies are important because they show a path from isolated devices to closed-loop functional systems. However, practical deployment still requires higher integration density, more efficient peripheral circuit design, lower system overhead, and stronger coordination among sensing, memory, processing, display, and power units. In this sense, the next step for neuromorphic memristors is not merely better device metrics, but compact and efficient full-stack hardware integration.

In the near term, research should prioritize standardized benchmarking protocols for device variability, endurance, retention, flexibility, and task-level evaluation, so that different memristor platforms can be compared on a common basis. In the medium term, advances in material engineering and manufacturable device structures should be combined with CMOS-compatible integration and stable array-level operation. At the same time, progress in yield control, process standardization, packaging compatibility, and technology transfer to scalable manufacturing lines will be essential for moving neuromorphic memristors from laboratory prototypes toward practical commercialization. In the long term, neuromorphic memristor systems are expected to evolve toward full-stack intelligent hardware that integrates sensing, memory, computing, and interaction, thereby supporting practical applications in wearable electronics, intelligent sensing, edge artificial intelligence, and human–machine interfaces.

Overall, Fig. 11 suggests that the future of neuromorphic memristors will depend on coordinated progress across multiple levels. Wearable and textile-based systems require scalable and standardized development, flexible devices require improved material and process control, in-memory architectures require more expressive brain-inspired computing models, and practical applications require efficient system-level integration. Therefore, future research should move beyond isolated performance demonstrations and focus more on the joint optimization of materials, devices, architectures, and intelligent system deployment.
